# Regulation of mitochondrial temperature in health and disease

**DOI:** 10.1007/s00424-022-02719-2

**Published:** 2022-07-02

**Authors:** Zaynab El-Gammal, Mohamed A. Nasr, Ahmed O. Elmehrath, Radwa A. Salah, Shams M. Saad, Nagwa El-Badri

**Affiliations:** 1 Center of Excellence for Stem Cells and Regenerative Medicine (CESC), Zewail City of Science and Technology, Cairo, Egypt; 2 Egypt Center for Research and Regenerative Medicine, Cairo, Egypt; 3 Faculty of Medicine, Cairo University, Cairo, Egypt

**Keywords:** Mitochondria, Mitochondrial temperature, Heat shock proteins, Oxidative phosphorylation, Uncoupling proteins

## Abstract

Mitochondrial temperature is produced by various metabolic processes inside the mitochondria, particularly oxidative phosphorylation. It was recently reported that mitochondria could normally operate at high temperatures that can reach 50℃. The aim of this review is to identify mitochondrial temperature differences between normal cells and cancer cells. Herein, we discussed the different types of mitochondrial thermosensors and their advantages and disadvantages. We reviewed the studies assessing the mitochondrial temperature in cancer cells and normal cells. We shed the light on the factors involved in maintaining the mitochondrial temperature of normal cells compared to cancer cells.

## Mitochondrial structure

Considered the powerhouse of eukaryotic cells, mitochondria are composed of mitochondrial matrix enclosed by an outer mitochondrial membrane (OMM) and intermembrane space enclosed by an inner mitochondrial membrane (IMM) [1, 4].

The structure of mitochondria is not always rigid. They form a highly dynamic network that responds to different stimuli, such as metabolic alterations and apoptosis [81].

Various types of cells require different mitochondrial demands; consequently, variations in mitochondrial structures are associated with multiple functional implications to meet the needs of each cell type. The cristae of the IMM are the sites of the protein complexes responsible for energy and reactive oxygen species (ROS) production [27]. Therefore, cells with greater energy demand have more cristae and less mitochondrial matrix volume. For example, heart cells display more cristae than matrix volume, while the opposite is true for liver cells [10, 58].

## Function of the mitochondria in normal cells

**Fig. 1:**
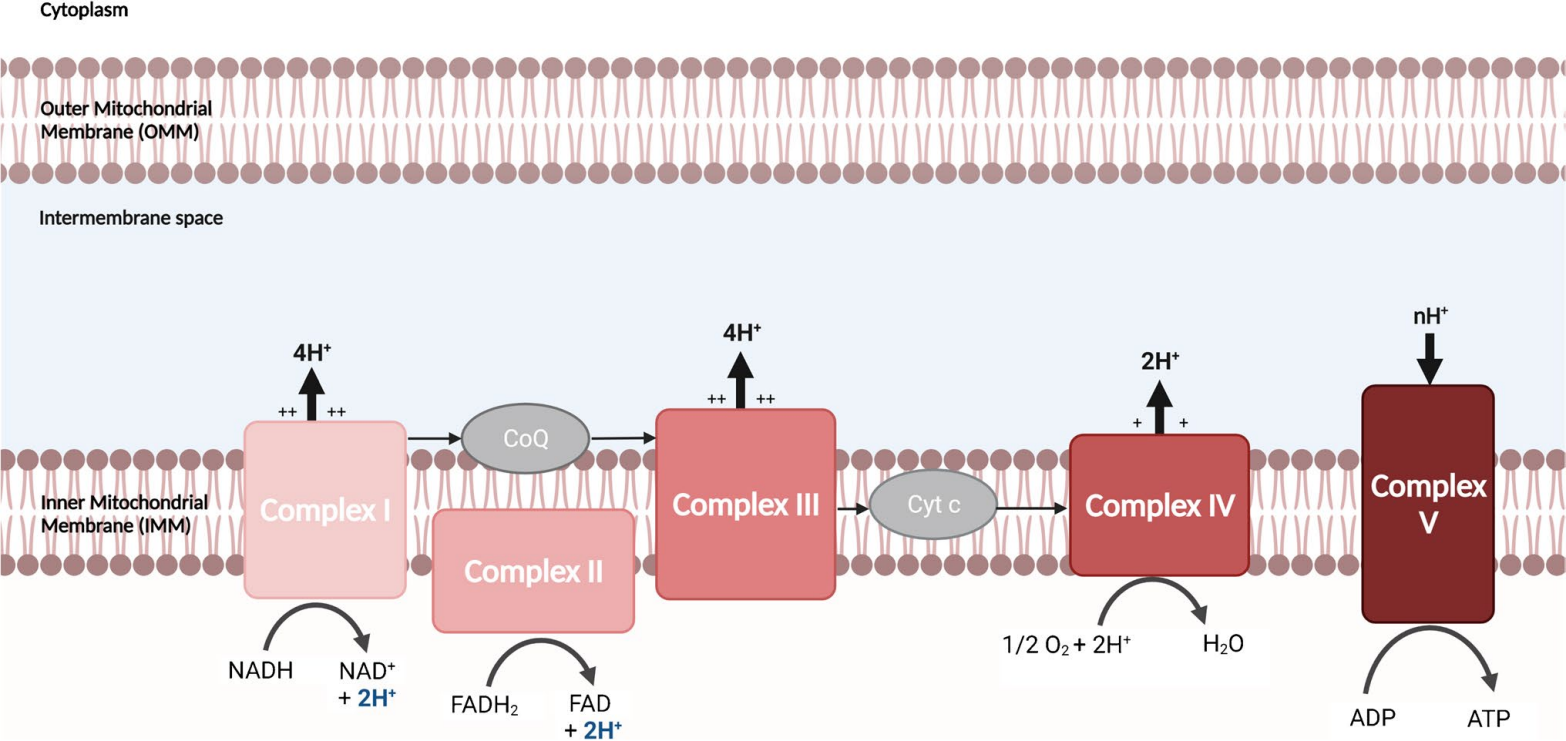
Electron transport chain transfer protons H^+^ across a membrane to synthesize ATP. *Created with BioRender.com*

Mitochondria have many critical functions in cellular metabolism. Five protein complexes (I to V), constitutively embedded in the IMM and known as super-complexes (SCs), are responsible for the oxidative phosphorylation (OXPHOS) process. Complex I (NADH-Q oxidoreductase) is responsible for NADH oxidation and proton transfer to the intermembrane space. Similarly, complex II (succinate dehydrogenase) oxidizes FADH_2_. Afterward, complexes I and II transfer their electrons to coenzyme Q (CoQ) (also known as ubiquinone), resulting in its reduction into ubiquinol. Ubiquinol then transfers its electrons to complex III (ubiquinol-cytochrome c oxidoreductase), which contributes to pumping more protons to the intermembrane space. Electrons from complex III are transferred to complex IV (cytochrome c oxidase) by cytochrome c (Cyt c) protein. Concomitantly, more H^+^ protons are transferred, and molecular oxygen is reduced into water. Finally, complex V (F_0_F_1_ ATP synthase) takes advantage of the developed proton motive force (PMF), in which the protons H^+^ re-enters through complex V to synthesize ATP from ADP [5], as shown in Fig. 1.

Electron cryotomography studies showed that the ETC protein complexes (I, II, III, IV) are found in less curved regions in the cristae, while F_0_F_1_ ATP synthases are found in tightly curved regions of the crista. Therefore, cells with high energy demand will have more tightly curved regions due to the importance of ATP synthases. For example, skeletal and cardiac muscles have more curved cristae than brown adipose cells to sustain their contractile ability [59]. On the other hand, brown adipose cells need less curved cristae due to the presence of more ETC complexes, since their energy is dissipated via a mechanism known as “uncoupling” [22, 37].

Mitochondrial functions are not limited to cellular metabolism or ATP production. Mitochondria initiate retrograde signaling to stimulate the nucleus to activate ATP production signaling pathways. This is achieved by activating the mitostress signals, via producing ROS and decreasing the inner mitochondrial membrane potential [33]. Also, mitochondria maintain cellular homeostasis by reducing ROS and H_2_O_2_ using antioxidant mechanisms, such as manganese-dependent superoxide dismutase and glutathione peroxidase [30, 55]. Importantly, mitochondria orchestrate the adaptive immunity of the cells by releasing mitochondrial antiviral signaling protein (MAVS), damage-associated molecular pattern protein (DAMP), and mitochondrial ROS [14, 48].

## Function of the mitochondria in cancer cells

For decades, cancer cells were thought to be defective in the mitochondrial respiration, and to undergo a high glycolysis rate to meet their energy requirements [28]. The dogma of cancer metabolism however has evolved, and today it appears that both glycolysis and mitochondrial metabolism are critical for tumor growth [78]. This is in accordance with Donnelly and Scheffler [24] who showed that cancer cells increase the rate of glycolysis and citric acid cycle (CAC) to maintain a constant amount of produced energy (∆*G*_ATP_).

Glycolysis replenishes the metabolites required for nucleotide synthesis and antioxidant activity through pentose phosphate pathway (PPP), NADPH production, nucleotide synthesis, and methylation reactions through one-carbon metabolism [21, 32]. Mitochondrial metabolism is crucial for tumor growth. Krebs cycle intermediates replenish the metabolites required for the synthesis of nucleotides, lipids, amino acids, and heme, all of which are required for the biosynthesis of new cells [82]. Cancer cells can replace the carbon used in the biosynthesis process by oxidizing glutamine to replenish α-ketoglutarate, branched-chain amino acid to replenish succinyl-CoA, pyruvate carboxylase to replenish oxaloacetate, and ubiquinol to replenish ubiquinone [20, 45]. This may reflect the importance of mitochondria for cancer cells to synthesize macromolecules and produce energy [83]. This was also confirmed by the importance of mitochondrial genome integrity and retention of function for cellular malignancy [36, 82]. This opposes the hypothesis of inefficient mitochondria in cancer cells as an explanation of Warburg’s effect that states that cancer cells shift their metabolism from oxidative phosphorylation to glycolytic pathways to meet their high energy demand.

## The importance of mitochondrial temperature

Different cellular dysfunctions and physiological and pathological conditions lead to altered energy metabolism and mitochondrial energetics [35], leading to a change in the mitochondrial temperature [69]. This is why mitochondrial temperature is an important indicator and differentiates between normal and dysfunctional cells [35]. One of the most prominent applications of mitochondrial thermometry is mitochondria-targeted drug delivery, using thermoresponsive nanocarriers. These nanocarriers are able to enhance the accumulation of anticancer drugs in mitochondria of specific endogenous mitochondrial temperature. This increases the selectivity to cancer cells and contributes to the reversal of drug resistance in cancer [63].

## Sources of mitochondrial temperature

**Fig. 2:**
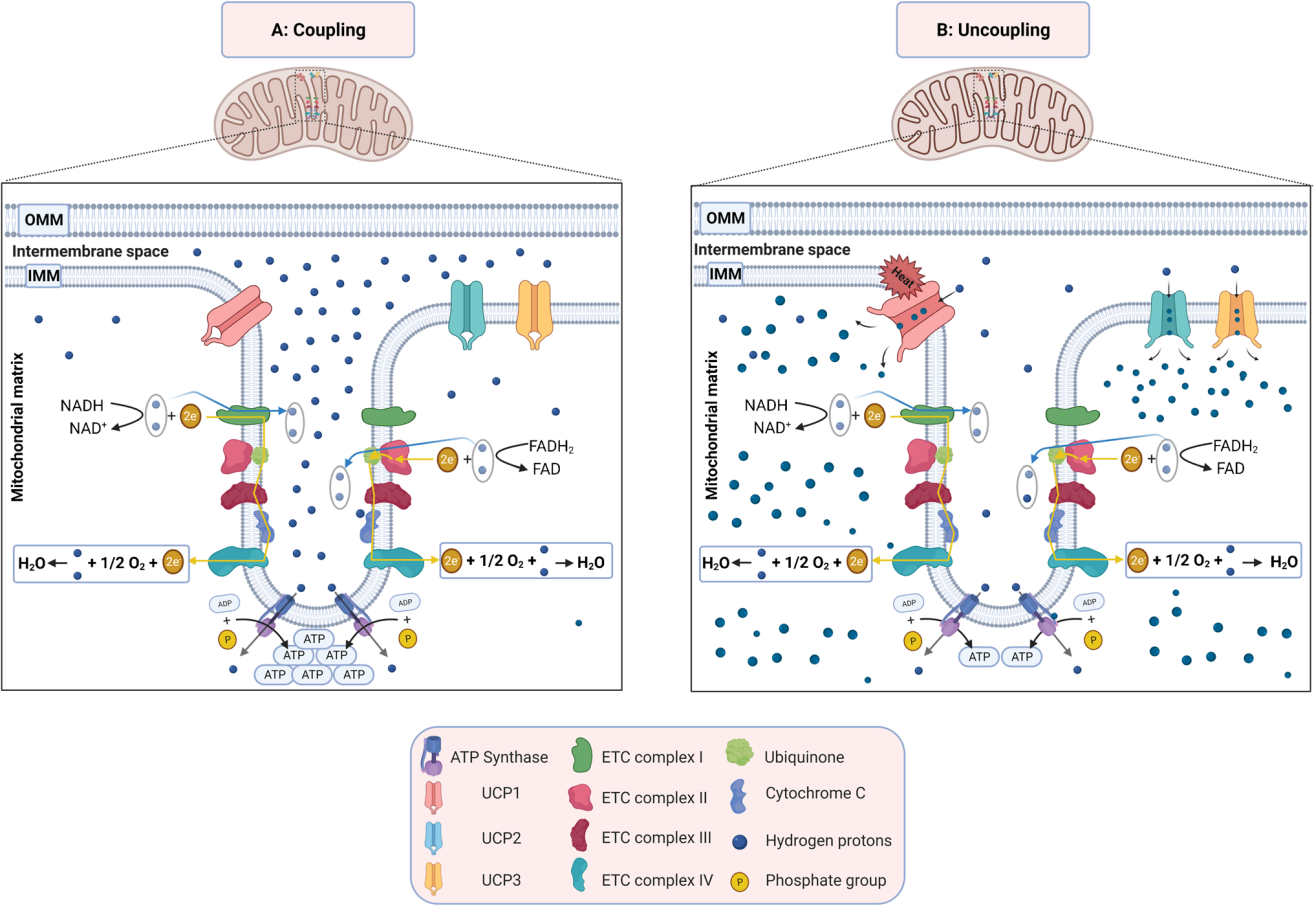
Mitochondrial coupling and uncoupling. **A** Mitochondrial coupling: proton pumps of the electron transport chain uses redox energy to generate proton motive force. This force will regenerate ATP by ATP synthase. **B** Mitochondrial uncoupling inhibits the coupling between electron transport and ATP-synthetic reactions. This in turn causes loss of the energy as heat. *Created with BioRender.com*

It was long believed that the primary source of mitochondrial temperature is mitochondrial uncoupling (Fig. 2B). ATP production is not 100% efficient since some H^+^ ions escape ATP synthase that converts ADP to ATP in the mitochondrial coupling, resulting in its return to the mitochondrial matrix via uncoupling proteins (UCPs) (Fig. 2). This process is the mitochondrial uncoupling that causes energy dissipation in the form of heat, and results in a transient increase in cellular temperature [2, 7, 8, 26, 40, 61, 67].

The increase in mitochondrial temperature due to uncoupling was shown upon treating human cervical cancer HeLa cells by carbonyl cyanide-4 trifluoromethoxy phenylhydrazone (FCCP) (mitochondrial oxidative phosphorylation uncoupler). The mitochondrial temperature increased from 32.6 to 35 °C with a concomitant decrease in the fluorescence intensity of the ATP sensing probe. This effect was abolished after removing FCCP [60]. It was also shown that there is a heterogeneity in the intra-cellular temperature, and that the mitochondrial temperature is no exception, as it follows the thermal diffusion equation [17, 38, 50, 64, 80].

## Methods to assess mitochondrial temperature

**Fig. 3:**
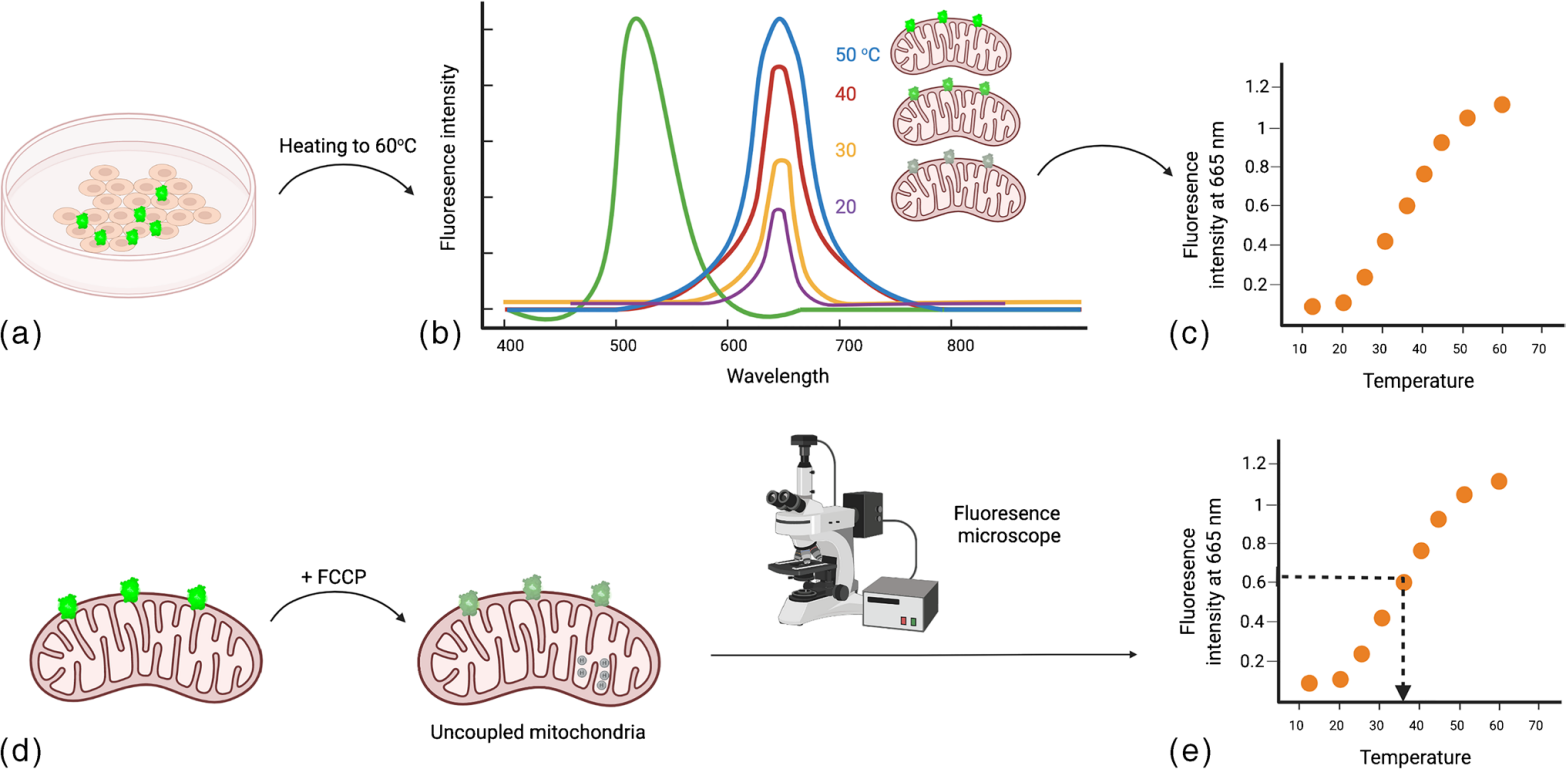
Measuring mitochondrial temperature by fluorescent thermosensors. **a** Cells are stained by fluorescent thermosensors and gradually heated. **b** Fluorescence intensity, measured by means of fluorescent microscopy at different temperatures will be recorded. **c** A plot of fluorescence intensity versus temperature will be drawn “Calibration plot.” **d** Uncoupler reagent (such as FCCP) will be added to induce mitochondrial uncoupling. **e** By means of fluorescence microscope, the fluorescence intensity of the cells will be measured by fluorescence microscopy and plotted (**c**) to conclude the cell temperature

Novel tools were considerably developed over the past decade to assess mitochondrial heat production, most notably by fluorescent thermometry. One of the ways to detect mitochondrial temperature change is to detect the effect of uncoupling on mitochondrial temperature. Uncouplers inhibit the coupling between electron transport and ATP-synthetic (phosphorylation) reactions. This in turn inhibits ATP synthesis, and instead, causes loss of the energy as heat [16, 70] (Fig. 3). A fluorescent nanogel was first used to detect an increase of ≈1 °C in African green monkey kidney fibroblast-like cells (COS-7) upon uncoupling [29]. The nanogel was later enhanced into a fluorescent polymeric thermometer (FTP), with higher accuracy and resolution. This was achieved using time-correlated single-photon counting system–based fluorescence lifetime imaging (FLIM) to image the localization of FTP in COS-7 cells, and estimate temperature change accordingly [53]. Although the average cellular temperature increased by around 1 °C, it was difficult to attribute the increase in temperature to mitochondrial heat production as the probes were localized in the cytoplasm, not specifically mitochondria [44].

Other fluorescent thermosensors localized to the mitochondria were developed by including rhodamine-CS-NIR-based Mito-RTP dye [34] and rosamine-compound-based MTY dye [3, 17]. Upon FCCP treatment of HeLa cells, Mito-RTP dye reflected an increase in mitochondrial temperature by an estimated 3.5–4 °C [34, 44]. Upon FCCP treatment, temperature sensing (7-(diethylamino) coumarin-3-carbaldehyde dye) and ATP sensing probe (rhodamine B derivative) reflected an increase in HeLa cells by 2.4 °C [60]. MTY showed an increase in mitochondrial temperature with increased respiratory activity in HEK 293 cells and primary skin fibroblasts, by up to 10 °C above that of the culture medium (38 °C) [17].

However, fluorescent thermosensors do not directly measure the temperature; they depend on the binding of the mitochondrial membrane (negatively charged) to the probe (positively charged) [60]. Thus, the change of the mitochondrial potential can change the localization of the dyes and can cause fluorescence quenching [18]. Also, fluorescent thermosensors are prone to biases in different cell types, metabolic status [39], and environmental conditions such as pH, viscosity, and ionic strength [44]. For example, Chrétien et al. [18] showed that MTY can measure the mitochondrial temperature of HEK and HeLa cells, but not the primary fibroblasts cells. This is why all data related to the environmental conditions must be reported, and purified probes must be used to study the effect of these parameters on the temperature measurement [44]. Moreover, the mitochondrial compartment in which the temperature was measured must not be extrapolated to the whole mitochondria without taking into consideration all the previous factors [18]. Thus, precise calibration in the extracellular environment and accurate colocalization of organelles with mitochondrial targeting dye are required for accurate mitochondrial temperature measurement [23]. Because of all the aforementioned limitations of these sensors, there is an urge to develop a stable and unbiased mitochondrial temperature assessment technique [18].

Other types of thermosensors that can localize inside the mitochondrial matrix are genetically encoded fluorescent proteins such as tsGFP [41], gTEMP [50], and emGFP [64]. When HeLA cells were treated with the uncoupler FCCP, fluorescence was detected by confocal, epifluorescence, and confocal fluorescence microscopy, respectively. tsGFP1 and gTEMP showed an estimated 6 °C increase in mitochondrial temperature [50], while emGFP reflected an estimated increase of 3–5 °C [44, 64]. Moreover, by means of these thermos-sensors, HELA cells’ mitochondria were shown to have different PMFs, and hence different intra-cellular temperatures. This feature is known as “thermal heterogeneity” and it has two levels: intra-cellular and inter-cellular. This is crucial for cell–cell behavior differences [6].

## Mitochondrial temperature in normal cells

Chrétien et al. [17] showed that the temperature of active mitochondria is 10℃ warmer than its normal physiological temperature due to the activity of respiratory chain (RC) complexes . Those were shown to reach their maximum potential at 48℃ in human embryonic kidney cells HEK 293, and in primary skin fibroblasts [17]. This observation was further confirmed by the inhibition of the RC complexes that lead to the decrease of the mitochondrial temperature from 50 to 38 °C [17]. In another set of experiments, Okabe et al. [53] measured the mitochondrial temperature of COS-7 cells using FTP and FLIM. They showed that the mitochondrial temperature was ranging from 43 to 48 °C.

The high mitochondrial temperature was attributed to the maximal respiratory chain complex activities [17]. This was supported by the enrichment of heat shock proteins and thermoprotectant solutes that protect the protein structures in high temperatures [18], and by the overexpression of the mitochondrial uncoupling protein UCP2 in liver cancer cells [11, 13, 15, 25]. This was confirmed by knocking down UCP2 expression in human hepatic cancer HEPG2 cells, leading to an increase in the mitochondrial membrane potential and ATP/ADP ratio, and a corresponding decrease in mitochondrial temperature [75]. Further experiments showed that peroxisome proliferator-activated receptor α (PPAR), the activator of UCP2, was overexpressed in liver cancer cells compared to normal liver cells [13, 46, 51, 56, 71].

## Mitochondrial temperature in cancer cells

As discussed previously, Warburg’s effect suggests that the cancer cells are defective in the mitochondrial respiration and must undergo high glycolysis in order to meet their energy supply requirements [28]. This implicates that the mitochondrial temperature in cancer cells is expected to be lower than that of normal cells. On the other hand, uncoupling is an important feature for cancer initiation, progression, metabolic adaptation, and drug chemoresistance [9, 73]. This implicates that the mitochondrial temperature in cancer cells is higher than that of normal cells.

Several reports confirmed that the mitochondrial temperature in cancer cells is higher than that in normal cells. Ruan et al. [63] showed that the mitochondrial temperature in murine breast cancer cells is about 48 °C. This was experimentally confirmed by showing that the highest release of the anticancer drug PTX loaded on thermoresponsive nanocarrier (poly N-isopropyl acrylamide, PNIPAM) was achieved when the lower critical solution temperature was adjusted to ~ 48^◦^C [62, 63]. This was assessed in murine breast cancer 4T1 cells at 48 °C compared to 37 °C and 25 °C [62]. Later, Wang et al. [76] also showed that the mitochondrial temperature was higher in murine cancer cells than in normal cells. The researchers used MTY dye staining of murine bladder cancer MB49 cells and murine RAW 264.7 macrophages. MB49 cells showed lower fluorescence of the mitochondria-targeted probe when compared with the RAW 264.7 cells, and correspondingly, a higher temperature. Moreover, Wang et al. [76] showed that the mitochondrial temperature in murine MB49 cancer cells was higher than that of human umbilical vein endothelial cells (HUVEC). However, we cannot find this conclusive due to species difference between the two cell lines.

Arai et al. [3] showed that the mitochondrial temperature of human lung carcinoma cell line H69AR was higher than that of HUVEC cells also using MTY dye [63]. However, Arai’s team was comparing malignant epithelial cells to normal endothelial cells and there was no proof that mitochondrial temperature difference is similar in both cell types. Importantly, the MTY assessment of mitochondrial temperature must be taken cautiously since, as previously mentioned, it is affected by the change in environmental factors and the affinity of the targets to the dye [18, 44]. The higher mitochondrial temperature in cancer cells on the other hand may be supported by the studies that showed that the mitochondrial respiration rate increases in cancer cells [74, 82].

**Fig. 4:**
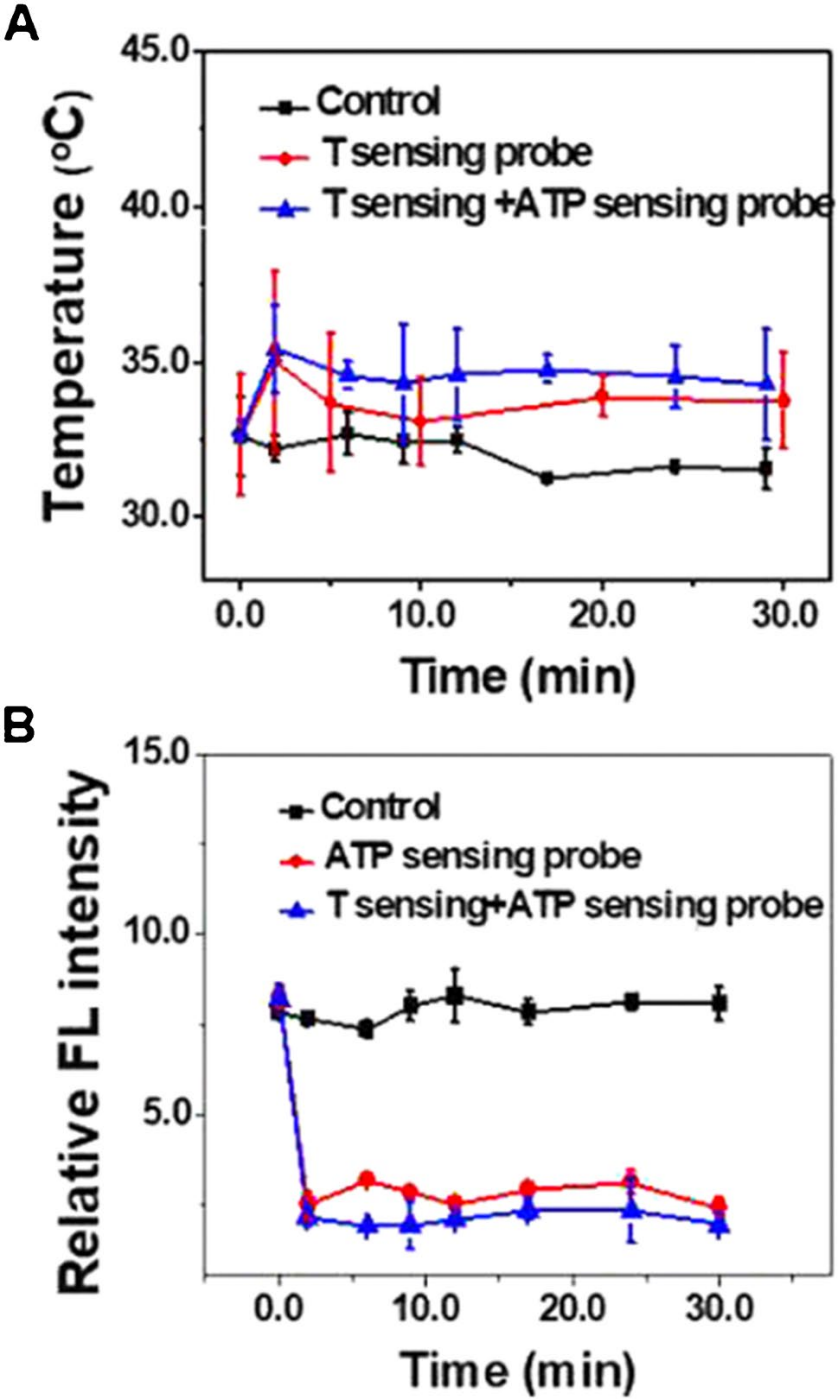
Mitochondrial temperature fluctuation response to FCCP inhibition in HeLa cells. Live HeLa cells were prestained with the T sensing probe (0.5 mg mL^−1^, 20 min) and the ATP sensing probe (5.0 μM, 20 min). The intensity data was obtained from 25 live HeLa cells. Reprinted with permission from “Qiao, J., Chen, C., Shangguan, D., Mu, X., Wang, S., Jiang, L., & Qi, L. (2018). Simultaneous monitoring of mitochondrial temperature and ATP fluctuation using fluorescent probes in living cells. *Analytical chemistry*, *90*(21), 12,553–12,558”, Fig. 6A. Copyright 2022 American Chemical Society

**Fig. 5:**
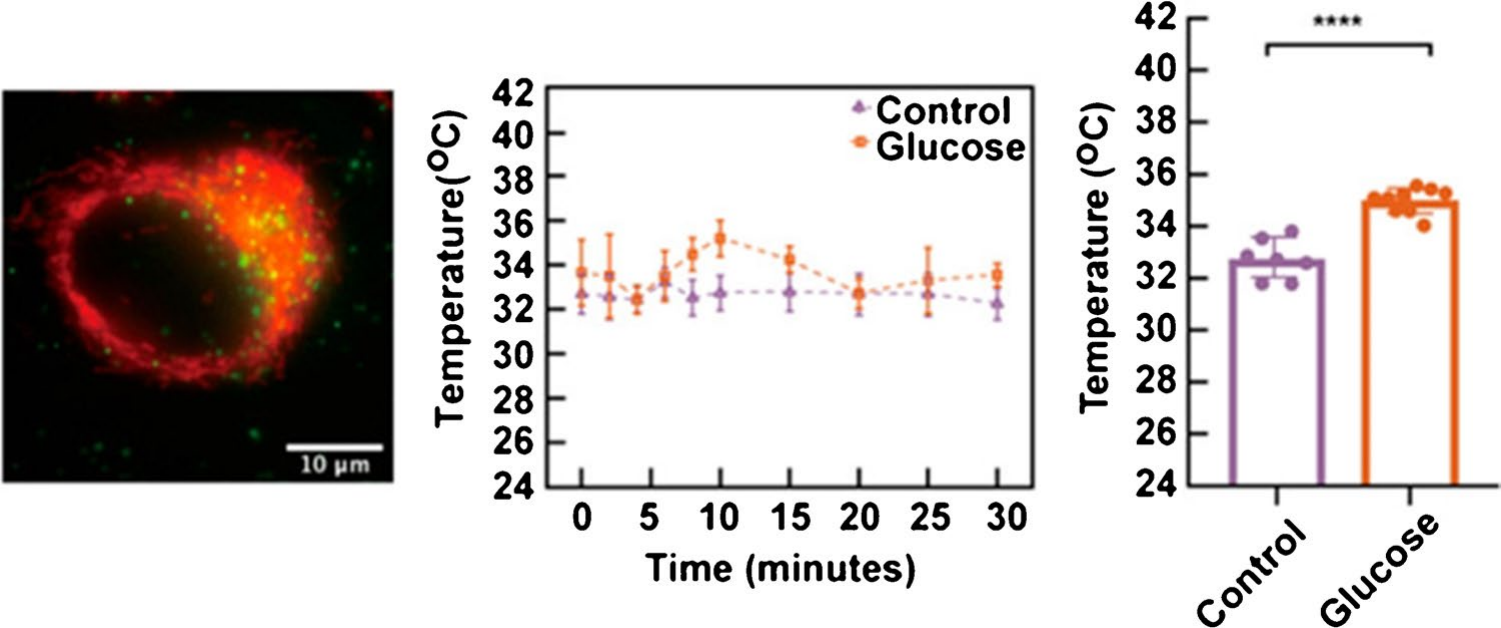
Visualization of mitochondrial thermal dynamics in HeLa cells response to glucose stimulations. (Left) Upconversion nanoparticles (UCNPs) at (3carboxypropyl) triphenylphosphonium bromide (TPP) images. MitoTracker (red) and UCNPs@TPP (green) from different treatments. (Middle) Mitochondrial temperature dynamics in the presence of 5 mg/mL glucose within 30 min. (Right) Student’s *t* test of both no glucose and glucose at 10 min (*p* < 0.0001). Reprinted with permission from “Di, X., Wang, D., Zhou, J., Zhang, L., Stenzel, M. H., Su, Q. P., & Jin, D. (2021). Quantitatively monitoring in situ mitochondrial thermal dynamics by upconversion nanoparticles. Nano letters, 21(4), 1651–1658”, Fig. 4A. Copyright 2022 American Chemical Society

Qiao et al. [60] measured the mitochondrial temperature in HeLA cells using PNIPAm-VBC-DACC-CTPP as a temperature sensor and RhB-ABA as an ATP sensor. From Fig. 4, we can conclude that HeLA’s mitochondrial temperature was 35–36 °C. Di et al. [23] assessed the mitochondrial temperature in HeLa cells using lanthanide-doped upconversion nanoparticles covalently linked to the mitochondria-targeting moiety (3 carboxypropyl) triphenylphosphonium bromide (TPP). From Fig. 5, we can conclude that the mitochondrial temperature of HeLa cells was 32–33 °C [23]. However, since these studies did not compare cancer to normal epithelial cells, there was no conclusive evidence that the mitochondrial temperature is higher or lower in cancer compared to normal cells. Indeed, comparing the mitochondrial temperature in those studies to that of normal epithelial cells in Chrétien et al. study suggests that the mitochondrial temperature in cancer cells is lower than that of normal cells. Still, caution must be taken since there is no standardized method to precisely measure the mitochondrial temperature, especially considering the limitations of MTY dye.

## Factors regulating mitochondrial temperature

### Mitochondrial membranes

Mitochondrial membranes play an important role in the regulation of mitochondrial temperature. The composition of the outer and inner mitochondrial membranes and their different protein-to-lipid ratios are primarily responsible for maintaining the mitochondrial temperature and providing layers of thermal insulation. The mitochondrial matrix is considered a narrow space flanked by heat-producing membranes to maintain the temperature difference between the cytosol and the mitochondria [17]. The cytosol contains mitochondrial domains to minimize heat conduction to the rest of the cell, as reported in HEK293 cells [17]. Recent research showed how the various composition of lipids in the different mitochondrial membranes affects the thermal stability of the RC complexes and the mitochondrial properties in response to temperature variation, such as swelling and permeability transition [17]. A study on liver mitochondria reported that the increase in membrane conductance due to hyperthermia was related to a sudden transformation in the order of the inner mitochondrial membrane [79].

### Heat shock proteins (HSPs)

Mitochondrial HSPs (mt-HSPs) were proposed to protect the mitochondria and keep their integrity under high mitochondria temperature [17, 52]. Seventy kDa HSP family (mt-HSP70) is remarkably abundant in the mitochondria of prokaryotes and eukaryotes [43]. HSPs are thought to play a role in protecting mt-DNA at relatively high temperatures [52]. This is extremely important since mt-DNA is not protected by a coat of histone proteins, rendering it more vulnerable to environmental insults such as heat and ROS damage [31]. This was also confirmed by the loss of mt-DNA function using mutant *S. cerevisiae* that do not express mt-HSP70 or mt-HSP78 [47]. Similarly, mt-HSP70 and mt-HSP40 contribute to maintaining mt-DNA structure and replication in human-pathogenic protist *Trypanosoma brucei* and yeast strains [19, 72].

Indeed, HSPs contribute to thermotolerance and thermoregulation by decreasing the thermally induced ROS oxidative damage especially that mitochondria are the main source of ROS [12, 31, 49, 52, 54, 68]

### FMC1 gene

The formation of the mitochondrial complex V assembly factor 1 homolog (FMC1) gene plays a critical role in maintaining the thermal stability of ATP synthase in yeast. This is accomplished by retaining the assembly and stability of the F1 part of ATP synthase, responsible for ATP synthesis, at high temperatures [42, 57, 65, 77]. C7orf55 is the homolog of the FMC1 gene in humans. Further research is needed to assess the extent of its contribution in maintaining the thermal stability of ATP synthase in human cells.

## Conclusion

Mitochondrial temperature is an essential indicator of mitochondrial function. Credible data suggest that cancer mitochondrial temperature is higher than that of normal cells. This finding can be of significance in the development of cancer biomarkers, specific anticancer drug delivery, and drug release from their conjugates. However, there is an urge to standardize the methods of measuring the mitochondrial temperature to be able to compare the results of different studies. Still, more comparative studies between normal and cancer cells of the same types are required to be able to identify whether the mitochondrial temperature can be used as a cancer biomarker.

## Data Availability

All data in this study are included in this manuscript.
